# Prevalence and Characterization of Dental and Skull-Bone Pathologies of the Raccoon Dog (*Nyctereutes procyonoides*) in Lithuania

**DOI:** 10.3390/ani13152437

**Published:** 2023-07-27

**Authors:** Eugenijus Jurgelėnas, Indrė Jasinevičiūtė, Linas Daugnora

**Affiliations:** 1 Department of Anatomy and Physiology, Veterinary Academy, Lithuanian University of Health Sciences, Tilžės 18, LT-47181 Kaunas, Lithuania; indre.jasineviciute@lsmu.lt; 2 Institute of Baltic Region History and Archaeology, Klaipėda University, Herkaus Manto 84, LT-92294 Klaipėda, Lithuania

**Keywords:** Canidae, raccoon dog, teeth, non-dental pathology, maxilla, mandible

## Abstract

**Simple Summary:**

The raccoon dog, a species introduced in Lithuania in 1950, has become an invasive species in the Baltic countries and has established itself as one of the prominent carnivores in the region. The primary objective of this study is to identify and compare dental and skull-bone pathologies between male and female raccoon dogs. The predominant pathology observed was tooth absence, with the majority of cases involving the absence of the mandibular third molar. Additionally, various stages of inflammation in the tissues surrounding the teeth were also noteworthy pathologies. Furthermore, our findings indicate that males exhibit a significantly higher prevalence of dental and skull-bone pathologies compared to females.

**Abstract:**

The present investigation endeavours to discern dental and non-dental pathologies affecting cranial structures of raccoon dogs, while focusing on cases of periodontitis. Furthermore, the study aims to conduct a comparative analysis based on sex and the nature of the pathologies encountered. The number of investigated skulls amounted to 126, including 76 males and 50 females. The predominant pathology identified was hypodontia, which accounted for 26.7% of males and 20% of females. Notably, the majority of hypodontia cases involved the absence of the mandibular third molar. Another noteworthy pathology was various stages of periodontitis, with rates ranging from 21.3% in males to 8% in females. Other pathologies, like tooth fractures and abrasion, were significantly less encountered. Excessive bone formation was relatively abundant and localized in specific areas—the parietal bone and the occipital regions. This tendency was observed in 8% of male cases and 6% of females. We found that the total number of dental and skull-bone pathologies is significantly more common in males than in females (*p* = 0.003). Additionally, the total number of various cases of periodontitis is more common in males too (*p* = 0.04).

## 1. Introduction

The raccoon dog (*Nyctereutes procyonoides*), an invasive species introduced in Lithuania in 1950, has established a significant presence following migration from Belarus and Latvia. Presently, the raccoon dog stands as one of the prevailing carnivores in the Baltic countries [1]. The native range of this species extends from northern Indochina through the eastern provinces of China and the Korean Peninsula to the south-east corner of Russia and Mongolia [2]. Maximum life span is seven to eight years, with a record in captivity of 13 years. Only about 1% of raccoon dogs live to five years, and 88% of the young (in Finland) die before their first year [3]. Morphologically, raccoon dogs exhibit a customary dental formula, as most other canids, characterized by the following dentition pattern: I 3/3, C 1/1, P 4/4, M 2/3 [3].

Pathologies of the teeth and periodontium of wild carnivores have been studied intensively [4,5]. Comprehensive investigations focusing on dental pathologies among members of the Canidae family have been conducted extensively in various European nations. Noteworthy studies of dental pathologies in red foxes have been researched in Poland [6] and the Czech Republic [7]. Moreover, similar studies were carried out on wolves in Latvia [8]. However, most of them include changes in the number of crown damage: abrasion, tooth fractures, and discoloration [9]. Previous studies lack pathologies of periodontal tissue of wild carnivores. Moreover, existing studies on dental pathologies often lack standardization and present incomplete methodologies [10]. In this study, a meticulous and structured approach was employed to evaluate dental and periodontal pathologies, facilitating expanded research even with limited sample sizes. Although wolf teeth pathologies were analyzed using the dental evaluation methodology, the author strongly recommends its application for assessing dental pathologies in other Canidae species [11]. Several skulls of representatives of the canine family have been studied using standard evaluation, including grey foxes [12], wolves [13], and kit foxes [14]. Notably, most of these studies were carried out in the USA.

Non-dental pathologies of the skulls of wild carnivores are poorly studied. Few studies examine the pathologies of wolves’ and coyotes’ skeletons (including skulls) [15]. A primarily methodologically detailed study of non-dental pathologies of lynx was carried out in Croatia [16]. Many studies are devoted to the changes in the shape of the foramen magnum in domestic dogs, including fossils [17], but such studies are scarce regarding wild carnivores.

The pathologies of the skeletons of representatives of the canine family are studied in Lithuania by the authors of the present article. Recently, we conducted a detailed study of teeth and non-dental pathologies in red foxes [18]. Since raccoon dogs breed in a limited area, their dental and non-dental pathologies are studied very poorly, especially in Europe. Even though the dental pathologies of Japanese raccoon dogs were analyzed, the mentioned subspecies do not live in Europe [19]. Available sources show that a preliminary study was carried out in Latvia where only 35 skulls of raccoon dogs were used, and only the changes in the number of teeth and crowns were examined [20].

This study conducted by our research team forms part of an ongoing series of investigations. The primary objective of this particular study was to examine both dental and non-dental pathologies among all members of the Canidae family residing in Lithuania. Additionally, our intention is to consolidate and synthesize the findings from these studies in the future, allowing for comparative analysis with data obtained from other countries.

The present study aims to identify the pathologies of the teeth, periodontium, and skull bones in males and females of raccoon dogs and to compare the obtained data by sex and the type of pathologies found.

## 2. Material and Methods

The study material was used from the Kaunas Tadas Ivanauskas Museum of Zoology collection of skulls of raccoon dogs (*Nyctereutes procyonoides*). Skulls were collected in the territory of the Republic of Lithuania from 1955. Sources of the collection were hunters and expeditors who found cadavers of animals. The collection of skulls is inventoried and has a registration card with the following parameters of the body: length, weight, and sex. In males, the penile bone (os penis) was attached with a registration card to confirm sex. Only the skulls of adult raccoon dogs were evaluated in the research. The maturity of the raccoon dogs’ skulls was determined by the closure of the presphenoid–vomer and basisphenoid–presphenoid sutures [12].

A total of 126 skulls and mandibles were included in this investigation, comprising 76 males and 50 females. The study comprehensively assessed and documented pathological alterations across various anatomical positions of the skulls. These pathological changes were meticulously described and captured through photography for thorough examination and analysis.

The methodology employed for evaluating dental conditions in this study was adapted from the approach described by Janssens et al. (2016) [11]. Specifically, the description provided by Evenhuis et al. (2018) [12] served as the foundation for assessing abrasion/attrition, periapical lesions, and also the temporomandibular joint pathologies. Notably, we excluded stage 1 of abrasion/attrition because the enamel fracture in the prepared skulls is potentially artefactual. Bone fractures and excessive bone formation were analyzed based on *Jubb, Kennedy & Palmer’s Pathology of Domestic Animals* [21]. Meanwhile, the investigation into changes of shape of the foramen magnum relied on the insights presented in *Miller’s Anatomy of the Dog* [22], and Janeczek et al. (2011) [23]. The categories encompassing all pathologies are presented in Table 1, providing a comprehensive overview of the pathological conditions.

## 3. Results

### 3.1. Dental Pathologies in Male Raccoon Dogs

**Figure 1:**
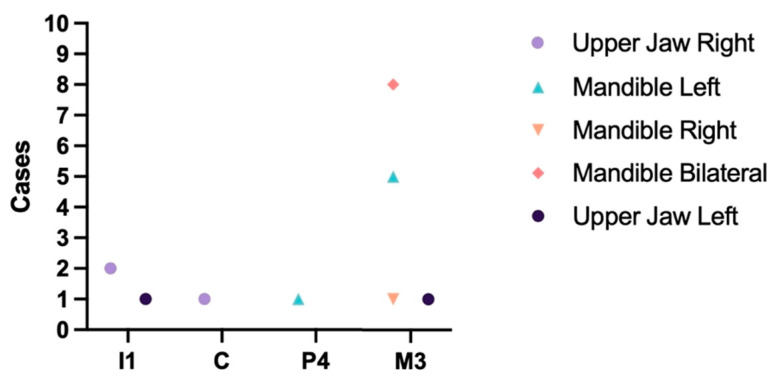
Distribution of cases of hypodontia in males. Blue triangle—Mandible left (Figure 2).

An abnormal number of teeth. Pathologies related to the number of teeth were investigated, with no inclusion of hyperdontia identified in the sample. Only a single case of traumatic dental lost (left canine tooth) was detected. Details regarding cases of hypodontia are presented in Figure 1, which also provides information on their distribution in the maxilla and mandible.

Tooth fractures. No instances of uncomplicated crown (or crown-root) fractures were detected in the study sample. However, a total of eight cases of complicated crown fractures were identified. Among the affected teeth, the majority were upper incisive teeth (five cases), and also canine and premolar teeth. It should be noted that questionable cases of dental fractures, which may arise during skull preparation, were excluded from the analysis.

Attrition/abrasion. Among the observed cases, a total of seven cases of various stages of abrasion were identified. Specifically, four cases were categorized as stage 2 abrasion, two cases as stage 3, and one case as stage 4. The canine teeth were the most commonly affected. Additionally, a case of stage 3, and one case of stage 4 were multiple abrasions of the upper and lower premolar and molar teeth.

No instances of enamel hypoplasia were detected in the study sample. Moreover, no cases of periapical lesions were found in the examined specimens

**Figure 3:**
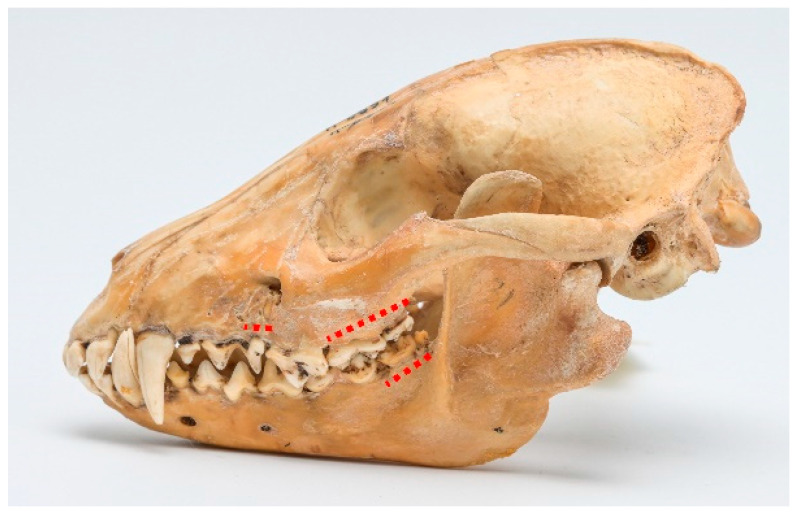
ID M–0951, male. Approx. length—120 mm. Second-stage periodontitis in the molar and premolar regions in the maxilla and mandible of the raccoon dog (marked with a dashed red line).

**Figure 4:**
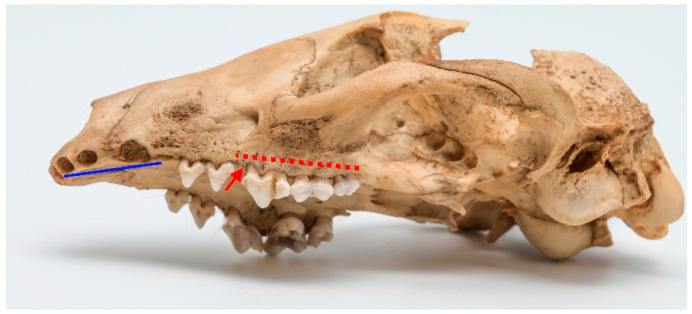
ID MP–2704, male. Approx. length—120 mm. Third-stage periodontitis with local dehiscence (marked by red arrow) in the molar and premolar regions of the raccoon dog (marked with dashed red line). Note: the blue line marks the artefactual teeth loss.

Periodontal disease. A total of 16 cases of various stages of periodontitis were observed. Stage 2 periodontitis was the most prevalent, with 13 cases (see Figure 3). Also, two cases of stage 3 periodontitis were detected (see Figure 4). The typical affected regions included the maxilla, ranging from P^3^ to M^2^, and the mandible, spanning from P_4_ to M_3_.

**Figure 5:**
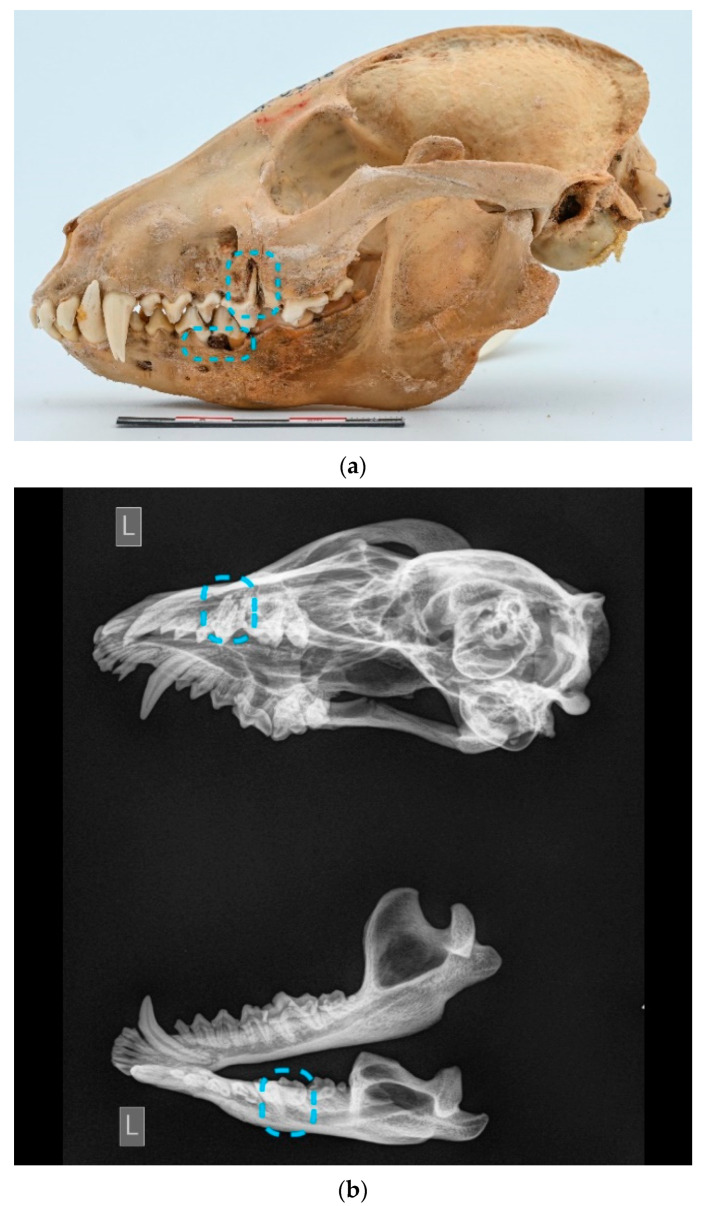
ID M–0948, male. (**a**) Approx. length—120 mm. Fourth-stage periodontitis with local dehiscence (marked by a blue square) of the left mandible and maxilla in the skull of the raccoon dog. (**b**) X-ray of fourth-stage periodontitis with local dehiscence (marked by a blue square) of the left mandible and maxilla in the skull of a raccoon dog.

Among observed cases, only one clear instance of stage 4 periodontitis was detected (ID M–0948; Figure 5a,b). The periapical bone resorption had developed around the length of the buccal root of P^4^. Furthermore, severe periodontitis characterized by significant bone resorption was observed around P^3^ as well. A milder stage of periodontitis was identified around I^3^, C, M^1^, and M^1^ of the left maxilla.

In the left mandible, both P_3_ and P_4_ exhibited pronounced effects of late-stage periodontitis, with the extent of bone resorption in the periapical area surrounding the roots of these teeth. The molar teeth on the left mandible displayed a comparatively lower degree of periodontitis, yet some loss of surrounding bone was evident.

Non-dental pathologies in male raccoon dogs. Fractures of skull bones and atypical shapes of the foramen magnum were not detected in any of the examined specimens. Two skulls were affected by a mild stage of temporomandibular joint osteoarthritis, while no moderate or severe cases were observed. Excessive bone formation pathologies were the predominant category in this group (Table 2). A total of six cases were identified, and each case will be described individually.

**Table 2:** Description of excessive bone formation pathological findings in males.

ID	Pathological Findings
M–0798	Spike-like osteophytes on external sagittal crest and temporal line.
M–0947	Small osteophytes on the nuchal crest, partial disappearance of the right side of the nuchal crest.
M–0943	Osteophytes on left side of zygomatic arch.
M–0792	Small osteophytes on external sagittal crest concentrated caudally (Figure 6).
M–1224	Osteophytes on external sagittal crest, caudal aspect.
M–2438	Osteophytes on nuchal crest, left side more concentrated.

**Figure 7:**
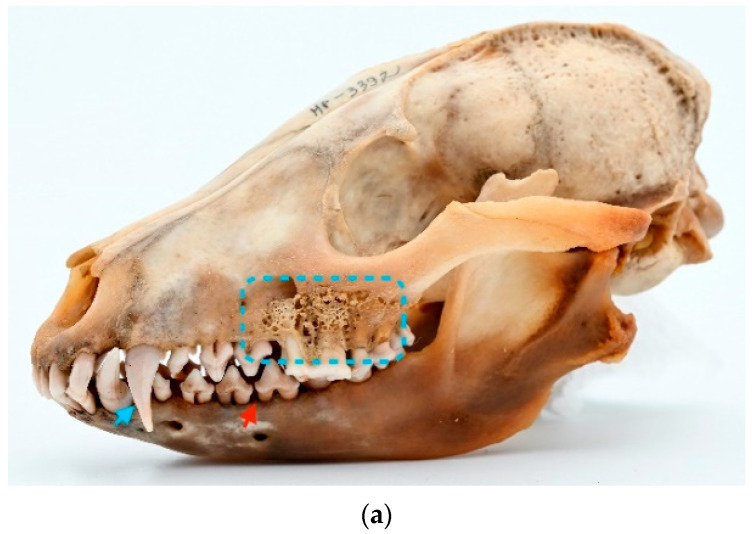

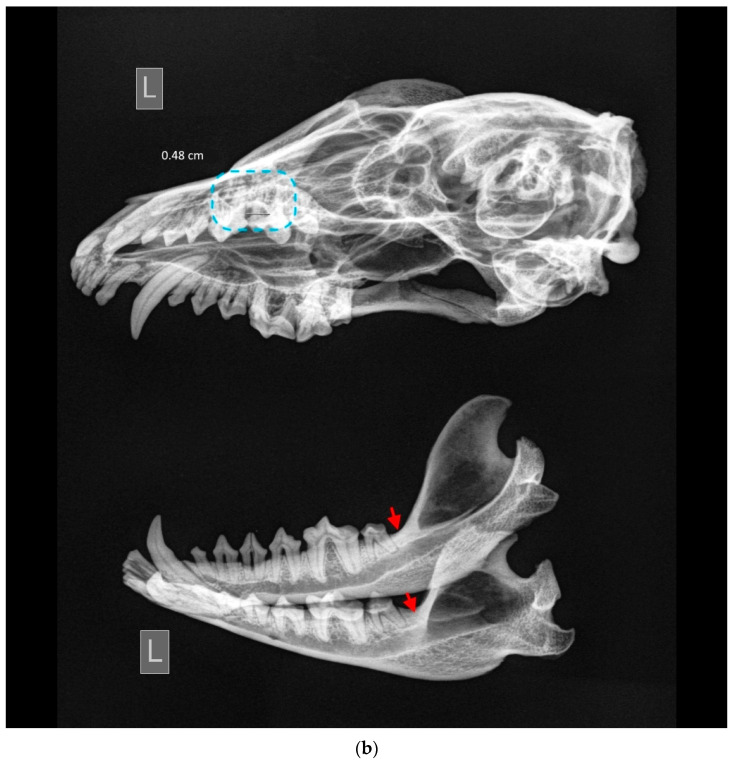
ID M–3392, male. (**a**) Approx. length—120 mm. Third-stage periodontitis with complicated horizontal dental fracture and periapical bone resorption (marked by a blue square) in the raccoon dog’s left side of the maxilla. Blue arrow shows the attrition of the left upper canine tooth, and red arrow shows the mild alveolar resorption. (**b**) X-ray of third-stage periodontitis with complicated horizontal dental fracture and periapical bone resorption (marked by a blue square) in the raccoon dog’s left side of the maxilla. Red arrows show the absence of the lower third molar tooth. No tooth buds were found.

Other findings. One particular skull (ID M–3392; Figure 7a,b) is severely affected and includes a lot of various kinds of dental and non-dental pathologies. This case warrants a detailed description of its specific findings. An absence of third lower molar teeth was detected. Complicated horizontal dental fracture with necrotic pulp and severe periapical alveolar resorption is present in P^4^ and rostral root of M^1^ of the left side of the maxilla. Furthermore, third-stage periodontitis with mild alveolar resorption affects all premolar and molar teeth, and attrition affects the left side’s lower and upper canine teeth. A similar pattern of periodontitis is evident on the left mandible. Moreover, multiple osteophytes had developed in the region of the zygomatic bone and external sagittal crest and nuchal crest.

### 3.2. Dental Pathologies in Female Raccoon Dogs

**Figure 8:**
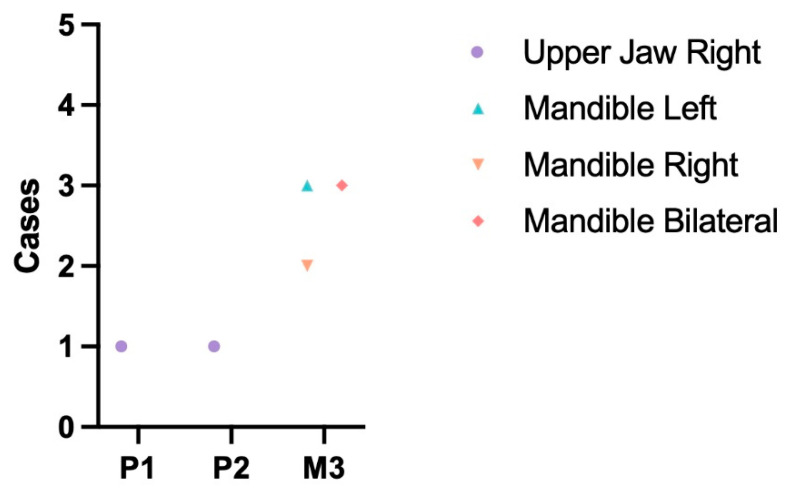
Distribution of cases of hypodontia in males.

An abnormal number of teeth. Figure 8 provides an overview of the findings in relation to dental numerical anomalies. Similar to the males’ skulls, no cases of excessive teeth were observed among the female specimens. Hence, the table exclusively displays cases of hypodontia. Furthermore, no traumatic dental-loss cases were detected in the female raccoon dogs.

Tooth fractures. Similar to the male specimens, no cases of uncomplicated crown fractures or crown-root fractures were found. However, two skulls exhibited complicated crown fractures. In one skull, the crown of left I1 and I2 was fractured, while the other one had fractures in both the left and right I1.

Attrition/abrasion. In total, we found eight cases of various stages of abrasion. Among them, five cases of stage 2 abrasion, one case of stage 3, and two cases of stage 4. The stage 2 abrasion primarily affected the caudal surface of the upper canine teeth. Additionally, one case of stage 3 and one case of stage 4 abrasion were founded on the upper and lower premolar and molar teeth.

**Figure 9:**
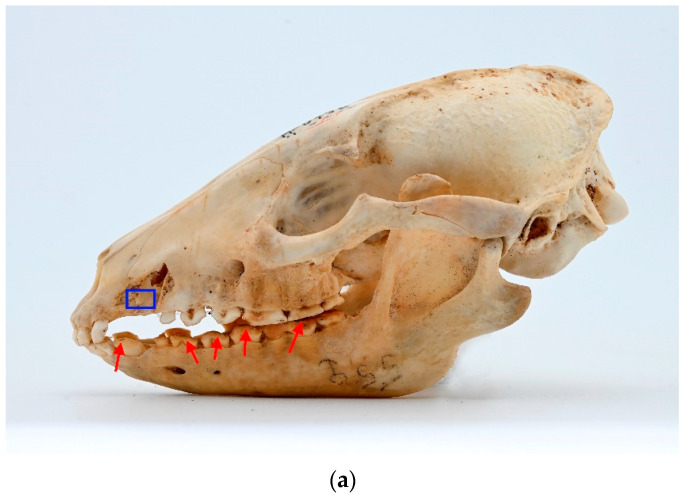

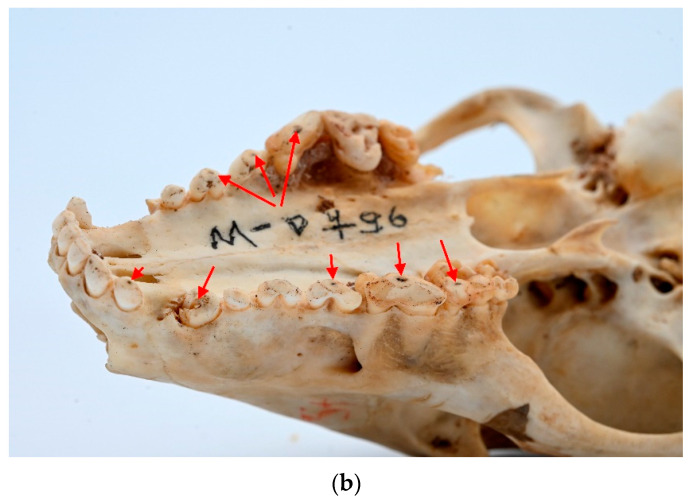
ID M–0796, female. (**a**) Approx. length—125 mm. Multiple abrasions (arrows) in the skull of the raccoon dog, lateral view. Note: blue square marks the artefactual tooth loss. (**b**) Approx. length—70 mm. Multiple abrasions (arrows) in the skull of the raccoon dog, ventral view.

Multiple teeth were affected in one skull (ID M–0796; Figure 9a,b). Almost all teeth were severely worn and were categorized as stage 4 abrasion.

**Figure 10:**
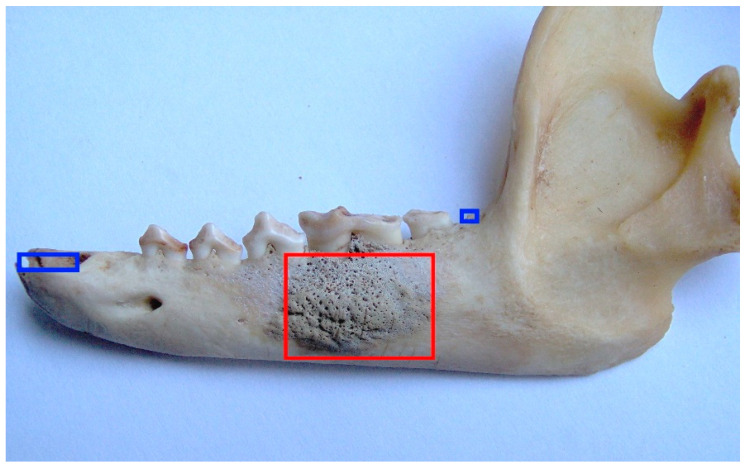
ID M–578, female. Approx. length—90 mm. Left-mandible porous bone tissue around the M_1_ (marked in red rectangle). Note: blue squares mark the artefactual tooth loss.

Enamel hypoplasia was not detected in the female raccoon dog skulls examined in this study. Regarding periapical lesions, only one case was identified (ID M–578; Figure 10). In the mandible, the bone tissue around the M_1_ was strongly affected, and resulted in porosity.

Periodontal disease. There were fewer cases of periodontitis in females’ skulls. In total, four cases of various stages of periodontitis were detected; from them, two cases of stage 2, and separate cases of stage 3, and stage 4 were detected. These cases of periodontitis were predominantly located in the maxillary region, spanning from P^4^ to M^2^.

Non-dental pathologies in female raccoon dogs. Non-dental pathologies in female raccoon dogs were relatively minimal. No bone fractures or atypical shapes of the foramen magnum were found. Additionally, no signs of temporomandibular joint changes were detected in the female skulls. Similar to the male skulls, the primary non-dental pathology observed was excessive bone formation, with three cases detected (Table 3).

### 3.3. Statistical Analysis

In total, 126 skulls with mandibles were examined, comprising 76 males and 50 females. Among the studied raccoon-dog specimens, a substantial proportion of 89 skulls (70.6%) exhibited various pathologies, including both dental and non-dental conditions. Specifically, the prevalence of pathologies was higher in males, with 61 cases (80.3%), compared to females, with 28 cases (56%).

The research findings revealed that pathologies of skulls in males were more common than in females (χ^2^ = 8.55, *p* = 0.003). A similar situation was revealed when we included dental and periodontal disease only (note: male skull ID M–3392 was excluded from the future statistics). Pathologies in males, 52 (69.3%), were more common than in females, 25 (50%) (χ^2^ = 4.74, *p* = 0.02). Non-dental pathologies in males, 8 (10.7%), were slightly more common than in females, 3 (6%), but there was no statistical significance (χ^2^ = 0.81, *p* = 0.36).

Distributions of dental and periodontal disease in the maxilla, mandible, and both jaws are presented in Table 4 Remarkably, pathologies affecting both jaws were more prevalent in males than in females.

## 4. Discussion

Hypodontia emerged as a prevalent pathology in our study, with isolated cases observed in both males and females. Only isolated cases of lack of incisors, canine, and molar teeth occurred in males and females, and hypodontia of the mandibular M_3_ molar tooth was the dominant one. This pattern should not come as a surprise since hypodontia of the mandibular M_3_ molar tooth is also very common in other representatives of the canine family. In our previous study, after examining 230 skulls of red foxes, we found that the lack of a mandibular M_3_ molar tooth was the most common compared to the lack of other teeth and accounted for 6.19% of cases in males and 7.69% in females [18]. A similar trend has been found in wolves. Of the 187 skulls studied, hypodontia accounted for 2.1%, of which M_3_ deficiency was the most common [8].

Hypodontia is a commonly observed condition in raccoon dogs, and its distribution pattern differs to some extent from other species. In Japanese raccoon dogs (*Nyctereutes procynoides viverrinus*), out of 179 skulls studied, hypodontia was found in 35. Similar to our findings, M_3_-molar-tooth deficiency prevailed, but another common hypodontia was a deficiency of both upper and lower teeth P1, which we found in our study only in one skull [19]. Interestingly, a significant disparity of results was found in the neighboring country Latvia where only one hypodontia was found in the 35 skulls of raccoon dogs studied. A lack of the premolar instead of the third molar was detected in the case [20]. Regarding the causes of hypodontia, we believe that all the cases identified in our study are of genetic origin rather than resulting from trauma. This implies that the affected teeth never erupted. In general, when it comes to hypodontia of M_3_, the loss of this tooth does not have a significant impact on survival. For carnivores, the canine, especially the carnassial teeth, are considered of primary importance [7]. We did not observe any lack of these critical teeth in our study.

In our study, only one case was described as a traumatic dental loss, specifically the loss of the left canine tooth in male skulls. No cases of traumatic dental loss were detected in the female skulls examined. It is worth noting that in other studies focusing on raccoon dogs, researchers usually do not separate dental losses into subcategories. However, when examining other animal species such as wolves, traumatic dental loss was detected more frequently. This pathology was detected in 9 skulls of 40 (22.5%) [11].

Our findings revealed that tooth fractures were observed in 10.66% of male skulls and 4% of female skulls. Unfortunately, there is limited information available regarding tooth fractures specifically in raccoon dogs from other countries. Research of other canids shows that tooth fractures are quite common. Yanagisawa et al. (2019) [14] found that tooth fractures in kit foxes (*Vulpes macrotis*) was found in 56.2% of specimens. Similar to our study, the dominant type was complicated crown fractures. In 187 grey-wolf skulls, Andersone and Ozolins (2000) [8] found that only two skulls had broken teeth. As in our study, incisors and canine teeth were the most common fractured teeth.

In contrast to many studies on teeth pathologies that often include cases of hyperdontia, our research did not identify a single case of hyperdontia in the examined raccoon dog skulls. Hyperdontia, the presence of an excessive number of teeth, was not observed in any of the specimens in our study. The same tendency was noticed in another study conducted in Latvia [20]. The trend is not unexpected since in skulls of other representatives of the canine family, only isolated cases of hyperdontia are detected. About 2% of the cases were found in red foxes [6]. However, in the studies examining raccoon dogs of another subspecies (*Nyctereutes procynoides viverrinus*), hyperdontia was quite common. In a study based on analyzing 179 skulls, hyperdontia accounted for 4.5% of teeth pathologies. In a similar study of 82 skulls, hyperdontia was identified in 6 skulls [19,24]. Asahara (2016) [25], who found that in 153 studied skulls of raccoon dogs, M_4_ extra tooth accounted for 2.61% of the pathologies, also gives a plausible explanation of the causes of hyperdontia. It is mainly associated with the high content of insects in the diet. The higher the range of insects, the larger the additional molars that are necessary for crushing chitin. Nutrition studies conducted in Lithuania showed that in the warm season, plants (raspberry, bilberry) and amphibians predominate in the diet of raccoon dogs. In the cold season, ungulate carrion (*Sus scrofa*, cervids), plants, and, in a smaller quantity, rodents make up the bulk of raccoon dogs’ diet [26]. To summarize, insects do not form a significant part of the diet in Lithuania.

As for damage to the surface of the dental crown, we found 7 (9.3%) cases tooth abrasion in males and 8 (16%) instances in females. Typical abrasion of the caudal side of the canine tooth and multiple premolars and molar teeth abrasions were found. Analogous cases are also detected in other species of canines. Abrasion of the exact nature were seen in our study in local red foxes [18]. The abrasion of the canine tooth can be explained by cage-biter syndrome, whereas the high content of sand can explain multiple abrasion of the teeth in food [11].

Periapical lesions were found to be rare in raccoon dogs based on our research findings. No individual cases of periapical lesions were found in the male skull, except in a severely affected skull (ID M–3392) together with another pathology. In females, only one skull had a clear case of periapical lesion. In other canids, the occurrence rate of this pathology is from only 0.4% in grey fox [12] to 5.3% in arctic fox [27].

Regarding periodontitis, our study revealed a significant difference in the number of cases between males and females. Males exhibited 16 cases of various stages of periodontitis, whereas females had only 4 cases. The predominant stage of periodontitis was stage 2, which was more common in males than in females. Stages 3 and 4 divide between sexes almost equally. The number of cases found in raccoon-dog populations in other countries remains uncertain, as there is a severe shortage of studies of periodontal diseases. Therefore, we must rely on similar studies of different species of the canine family. Evenhuis et al. (2018) [12] found that almost half of the specimens (n = 276, 48.7%) displayed some degree of bony change consistent with periodontitis after studying 569 of the skulls of grey foxes (*Urocyon cinereoargenteus*). Within the study, a group of 226 (39.7%) specimens displayed bony change suggestive of stage 2 periodontitis, 120 (21.1%) with stage 3, and 13 (2.3%) with stage 4. In our study, the number of detected cases of stage 2 periodontitis is much lower. The limitation of our study lies in the relatively smaller amount of material available. In addition, we included only obvious cases, while we rejected dubious ones. It has to be admitted that the diagnosis of stage 2 periodontitis when examining the bone material is complicated.

Similar to our previous study on red foxes, we also investigated non-dental pathologies in raccoon-dog skulls. In our previous study of red foxes, we found small numbers of varied pathologies, among them one bone fracture and two cases of an atypical form of the foramen magnum [18]. However, in the current study, we did not identify such pathologies. This should come as no surprise since both skull fractures and the atypical form of the foramen magnum is usually critical in terms of survival. The atypical condition of the foramen magnum adversely affects the spinal cord, resulting in neurological disorders [23].

Temporomandibular joint osteoarthritis is a rare pathology observed in the Canidae family. Evenhuis et al. (2018) [12] found that none of the specimens of grey foxes have any sign of TMJ osteoarthritis. In the kit foxes, 5.9% of the specimens have a sign of low-grade temporomandibular joint osteoarthritis [14]. In our study, TMP osteoarthritis occurs only in 2.7% of males and not at all in females.

In the present study, we found a wide variety of bone growths—osteophytes in specific areas of the skull, i.e., external sagittal crest, nuchal crest, and the zygomatic bone. These areas were affected in both males and females. Although there were more of these pathologies in the skulls of males than in the skulls of females, the difference was not statistically significant. Unfortunately, there are very few studies of non-dental pathologies. Gomerčić et al. (2009) [16] examined 58 skulls of Eurasian lynx (*Lynx lynx*) and identified non-dental pathologies only in 2 skulls. In this study, lesions were found in the area of the frontal bone, and only in 1 skull were there bony outgrowths that covered the frontal bone’s zygomatic process.

The interpretation of osteophytes in specific areas presents challenges. However, it can be hypothesized that the proliferation of osteophytes in the external sagittal crest and the nuchal crest is associated with the attachment sites of certain muscles. Evans and de Lahunta (2013) [22] propose that the external sagittal crest forms the medial boundary of the temporal fossa, a large area on the external surface of the cranium from which the temporal muscle originates. Consequently, the development of the osteophytes of the external sagittal crest can be attributed to the development of the temporal muscle and increased load.

The assessment of the differences between males and females did not reveal statistically significant differences in almost any category of pathologies, except for the total number of periodontitis cases, which was more common in males than in females (*p* < 0.05). A slight tendency for pathologies is more common in males than females, which is observed in hypodontia and tooth fractures. Also, pathologies are more commonly located in both jaws in males than in females (*p* = 0.005), but not in only maxilla or mandible. Consequently, it can be concluded that the prevalence of dental and non-dental pathologies in the population of raccoon dogs is not dependent on sex. This finding aligns with the results obtained from the examination of red-fox populations, where no statistically significant differences were found, except in the category of multiple pathologies [18]. This trend is observed in most other canine family studies. Szuma (1999) [6], who studied the pathologies of the teeth of red foxes, also did not find statistically significant differences in individual categories, although dental pathologies were generally more common in males. Similarly, in our study, we found that dental pathologies are more common in males than in females (*p* = 0.02).

## 5. Conclusions

In conclusion, the prevalence of dental pathologies in raccoon dogs in Lithuania appears to be comparable to findings from studies conducted in other countries. Admittedly, the lack of similar studies was significant, and the comparison of data had to be based mainly on data from studies of the Japanese subspecies. Hypodontia of the mandibular third molar tooth could be singled out, which is the most common pathology and coincides with the data of studies of most representatives of the canine family. Furthermore, a notable result of this study is the identification of an abundant but only singular dominant type of non-dental pathology. The observed increase in bone tissue proliferation is relatively significant in the occipital- and parietal-bone areas and prompts a more detailed study of the causes of these pathologies in the future.

## Figures and Tables

**Figure 2:**
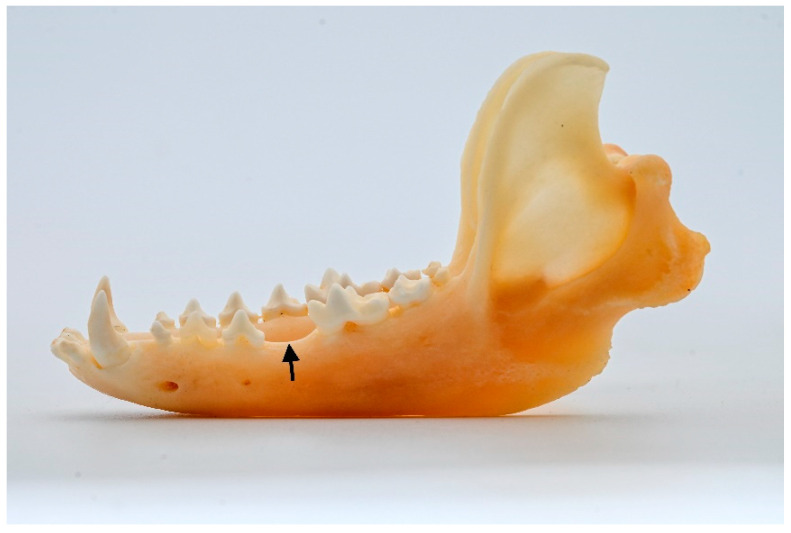
ID M–4688, male. Approx. length—90 mm. Absence of the P_4_ (arrow) in the left mandible of the raccoon dog.

**Figure 6:**
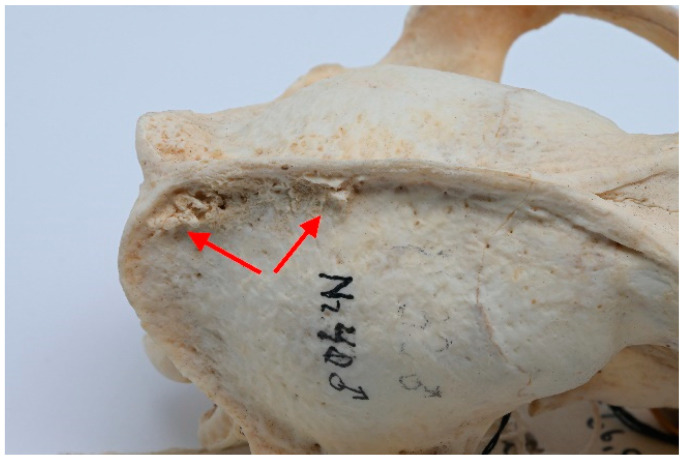
ID M–0792, male. Approx. length—65 mm. Osteophytes of the external sagittal crest (marked by red arrows).

**Table 1:** Descriptions of dental and non-dental pathologies.

Categories	Sub-Categories	Short Explanation
Dental pathologies		
Abnormal number of teeth	Hypodontia	No alveolus present (genetic)
	Traumatic	A tooth is missing, and the alveolus is visible and deformed
	Excessive teeth	Polyodontia/hyperdontia (genetic)
Tooth fractures	Uncomplicated crown (or crown-root) fracture	Fracture without pulp exposure
	Complicated crown (or crown-root) fracture	Fractures with pulp exposure
Attrition/abrasion	Stage 2	Exposure of dentine on the cuspal tip, without tertiary dentine formation
	Stage 3	Exposure of dentine on the cuspal tip, with tertiary dentine formation
	Stage 4	Pulp cavity exposure secondary to attrition/abrasion
Enamel hypoplasia	Mild	Brownish enamel discoloration
	Severe	Part of tooth is enamel free, dentine visible
Periapical lesions	–	Macroscopically visible periapical bone loss, root tip resorption, sinus tract formation originating periapically, or obvious focal periosteal reaction overlying the apex
Periodontal disease	Stage 2	Less than 25% loss of alveolar bone
	Stage 3	More than 25% but less than 50% loss of alveolar bone
	Stage 4	More than 50% loss of alveolar bone
Non-dental pathologies		
Temporomandibular joint (TMP) osteoarthritis	Mild	Evidence of early periarticular new bone formation/osteophytes with minimal or no subchondral bone change
	Moderate	Periarticular new bone formation and/or subchondral bone changes
	Severe	All previously described signs are present and more pronounced; subchondral bone lysis present; partial or complete ankylosis may be observed
Bone fractures	–	–
Excessive bone formation	–	Exostosis or osteophytes.
Shape changes of the foramen magnum	–	Deviations from the typical shape of the foramen magnum—oval or circular. A visible notch to any side is fixed as an abnormality.

To validate the observed pathological changes, a subset of cases underwent digital X-ray imaging. Digital X-ray imaging was conducted at a private small animal clinic Ltd. “VetPet LT”, using Amadeo V–AX stationary system. Descriptive studies were employed to examine data. For the comparison, the percentages were calculated. The statistical data analysis was performed using SPSS 24.0. Statistical comparison between males and females was evaluated via the Chi-square test. Statistically, the significant parameter was when *p* < 0.05.

**Table 3:** Description of excessive-bone-formation pathological findings in females.

ID	Pathology Description
M–0808	Small dot-like osteophytes covering almost all of the parietal bone.
M–615	Spike-like osteophytes concentrated on the external sagittal crest, particularly between the external sagittal crest and temporal line.
M–2165	Osteophytes on the external sagittal crest, with a higher concentration near the connection with the nuchal crest.

**Table 4:** Distribution of dental and periodontal pathologies in various parts of the skull.

Sex	Maxilla	Mandible	Located in Both Jaws
Males (n = 75)	16 (21.3%)	17 (22.7%)	19 (25.3%)
Females (n = 50)	13 (26%)	9 (18%)	3 (6%)
χ^2^	0.36 (*p* = 0.54)	0.39 (*p* = 0.52)	7.73 (*p* = 0.005)

The investigation of various types of dental and skull-bone pathologies, as summarized in Table 5, revealed only one statistically significant difference between males and females. Specifically, the overall prevalence of periodontal disease was found to be higher in males compared to females (*p* = 0.04).

**Table 5:** Statistical significance and distribution of pathologies.

Pathology	Males n = 75	Females n = 50	χ^2^
Hypodontia (total)	20 (26.7%)	10 (20%)	0.73; *p* = 0.39
Hypodontia—M^3^ only	14 (18.7%)	8 (16%)	0.14; *p* = 0.70
Traumatic tooth loss	1 (1.3%)	0	0.67; *p* = 0.41
Tooth fractures	8 (10.6%)	2 (4%)	1.81; *p* = 0.17
Abrasion (total)	7 (9.3%)	8 (16%)	1.26; *p* = 0.26
Periapical lesions	0	1 (2%)	1.51; *p* = 0.21
Periodontitis (total)	16 (21.3%)	4 (8%)	3.96; *p* = 0.04
Periodontitis (stages 3 and 4 only)	3 (4%)	2 (4%)	0.00; *p* = 1
Excessive bone formation	6 (8%)	3 (6%)	0.17; *p* = 0.67
TMP osteoarthritis	2 (2.7%)	0	1.35; *p* = 0.24

## Data Availability

Authors can confirm that all relevant data are included in the article and are available from the corresponding author upon reasonable request.
